# Just-World Beliefs, System Justification, and Their Relationship with People’s Health-Related Well-Being: A Narrative Review

**DOI:** 10.3390/bs14100941

**Published:** 2024-10-14

**Authors:** Camilo Silva, Víctor Pedrero, Jaime Barrientos, Jorge Manzi, Katiuska Reynaldos

**Affiliations:** 1 Faculty of Nursing, Universidad Andrés Bello, Santiago 8370146, Chile; victor.pedrero@unab.cl (V.P.); katiuska.reynaldos@unab.cl (K.R.); 2 Faculty of Health Science, School of Nursing, Universidad Católica Silva Henríquez, Santiago 8330226, Chile; 3 School of Psychology, Universidad Alberto Hurtado, Santiago 8340575, Chile; jbarrientos@uahurtado.cl; 4 School of Psychology, Pontificia Universidad Católica de Chile, Santiago 8331150, Chile; jmanzi@uc.cl

**Keywords:** just world, system justification, health, well-being

## Abstract

Beliefs about a social system help people understand and evaluate their environment and are related to their behavior within a society. When people believe that they live in a just social system and develop positive attitudes about the social and political environment, they experience greater satisfaction and well-being. This phenomenon is known as a palliative effect. Two theoretical approaches that explain support for social and political systems are the beliefs in a just world and system justification approaches. The objective of this review was to analyze the literature published between 2019 and 2023 that addressed the associations of beliefs in a just world and system justification with health-related well-being. The search yielded 2064 potentially eligible articles, 26 of which were ultimately selected. The results showed that beliefs in a just world are associated with a more positive perception of the world and better health outcomes. In addition, people with a greater propensity to justify the system experience higher levels of health-related well-being. This positive association is consistently observed across different social groups and contexts. These findings support the phenomenon of palliative effects attributable to beliefs in a just world and system justification.

## 1. Introduction

Well-being encompasses a wide range of aspects that go beyond physical health and include emotional, intellectual, and financial dimensions, as well as the perceptions and beliefs that people have about the social and political environment in which they live. These different facets are intertwined to determine the general state of well-being of a person [1,2].

Beliefs about a social system are important, as they help people understand and evaluate the world around them and relate to their behavior within a collective reality that involves social, political, cultural, and economic aspects [3]. Evidence has shown that beliefs about a social system can affect people’s experiences and perceptions of well-being [2,4]. When people believe that they live in a just social system and develop more positive attitudes about the social and political environment in which they live, they may experience a greater sense of satisfaction and well-being.

Two theoretical approaches that help in understanding the degree to which people support social and political systems are the beliefs in a just world and system justification approaches. There is a close relationship between these two ideological approaches, to the point that the system justification theory considers belief in a just world a relevant factor within its theoretical formulation [5,6,7,8]. Both ideologies support and justify the status quo within society; that is, they support the way in which a given society traditionally functions and the inertia of keeping the situation as it is without interference [5,6,9,10].

The beliefs in a just-world theory alludes to the idea that people within a society receive what they deserve and deserve what they receive [11]. These beliefs are divided into two categories: general and personal beliefs in a just world. The former category refers to the perception that people obtain results in accordance with what they deserve, whereas the latter category focuses on the conviction that society in general is fair [12,13]. General and personal beliefs in a just world are correlated with each other, which means that people who believe that others obtain what they deserve also tend to believe that they themselves obtain what they deserve [14]. It has been shown that, to the extent that people adhere to this belief system, they have a greater perception of well-being and have lower levels of psychological distress [15,16]. The benefit of this ideology would be derivative of its capacity to promote resilience. Resilience could be an important mechanism in the relationship between just-world beliefs and well-being, as it would allow individuals to face adverse situations with a more positive attitude [17].

On the other hand, the system justification theory corresponds to a set of beliefs through which people tend to legitimize and rationalize the existing structures and hierarchies in society [6]. System justification theory postulates that people have an intrinsic motivation to perceive the social system in which they live as fair and legitimate; this motivation would stem from its capacity to satisfy epistemic, relational, and existential needs [7]. When people perceive the social system in this way, they experience a sense of order, stability, and security in their environment, which generates feelings of satisfaction and well-being [8]. Research in this line has shown that those people who justify the social system in which they live would experience a lower perception of stigma and greater self-esteem and life satisfaction [18,19,20].

The positive association between these ideologies that support the status quo and well-being is known as the phenomenon of a palliative effect [10,21]. In the system justification theory, the palliative effect is defined as a phenomenon in which people, by justifying or believing in the existing social system, experience emotional or psychological relief with respect to their personal situation, even if that situation implies disadvantages or inequalities. This palliative effect suggests that the acceptance of the status quo would provide comfort or reassurance to people, which may lead them to feel better about their position in society, even if it is not favorable [21,22,23,24].

Some authors have conducted literature reviews that explore the relationship between beliefs in a just world or system justification and people’s health-related well-being from the earliest postulates up to the year 2019 [25,26,27]. The objective of this narrative review of the literature was to analyze the recent literature on how the ideologies of beliefs in a just world and system justification are associated with people’s health-related well-being.

## 2. Methods

A narrative review of the literature was carried out for recent articles that addressed the association of beliefs in a just world and system justification with people’s health-related well-being. This study was carried out according to the Preferred Reporting Items for Systematic Reviews and Meta-Analyses (PRISMA) recommendations [28].

A search of the Web of Science (WoS) and Scopus databases was carried out with the keywords health, well-being, anxiety, mental health, physical health, quality of life, stress, self-esteem, life satisfaction, just world, and system justification. These terms were combined using the Boolean logic operators AND and OR (Table 1).

Articles with a focus on any of the aforementioned ideologies and well-being published between 2019 and 2023 were selected. No language or design restrictions were applied. Meta-analyses, scoping reviews, and literature reviews on any of the ideologies mentioned were excluded.

**Figure 1:**
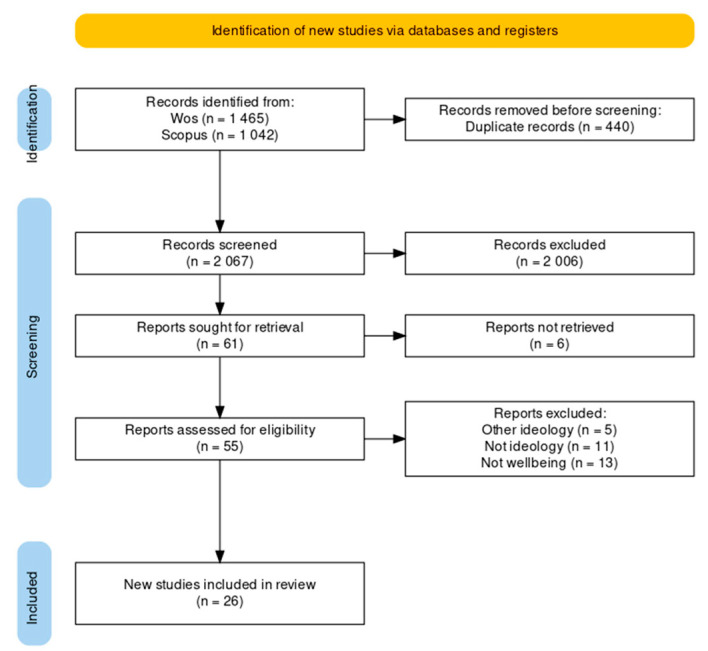
PRISMA2020 flow diagram for eligible articles. Note: own elaboration.

To select articles, first, the titles and abstracts of the articles were screened. Then, the full text of each article was read, and articles were selected if they met the predefined inclusion and exclusion criteria. All duplicate articles, articles for which the full text was not available, and articles that did not meet the inclusion criteria were excluded. To facilitate the understanding of the methodology used for article selection, a PRISMA2020 flow diagram was developed [29] (Figure 1).

For data extraction, a standardized and predefined registration form in Microsoft Excel (version 16.73 for Mac) was used, which included title, year, country, objective, design, population, ideology, measurement scales for ideology and health-related well-being, results, and conclusions.

## 3. Results

Twenty-six articles were included in this review, most of which were from Asia (39%, *n* = 10), followed by Europe (23%, *n* = 6), Oceania (19%, *n* = 5), North America (15%, *n* = 4), and South America (4%, *n* = 1). With respect to ideologies, 61% (*n* = 16) of the studies address beliefs in a just world, whereas 39% (*n* = 10) address system justification. With respect to the study population, ten studies address ideologies and their relationship with health-related well-being in the general population (39%), nine studies address adolescent students (35%), three studies address women (11%), three studies address sexual minorities (11%), and one study addresses persons deprived of liberty (4%). Eighty-five percent (*n* = 22) of the selected studies are cross-sectional studies, and fifteen percent are longitudinal studies (*n* = 4). The characteristics of the 26 articles selected for this narrative literature review are summarized in Table 2.

The main findings of the studies that involve beliefs in a just world, system justification, and their association with health-related well-being are presented below.

### 3.1. Beliefs in a Just World and Health-Related Well-Being

**Figure 2:**
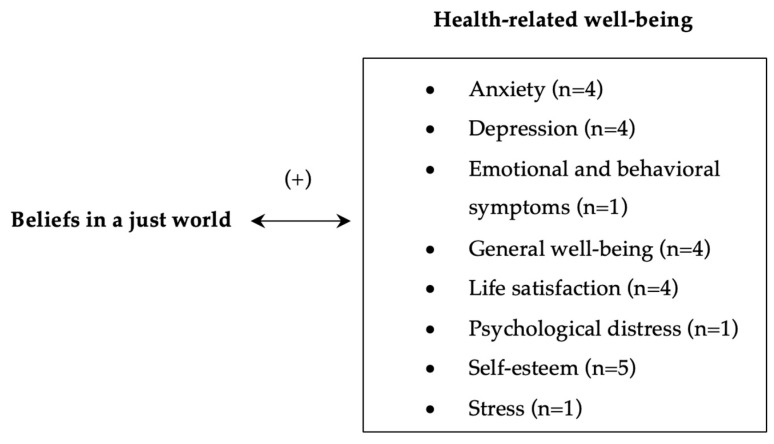
Beliefs in a just world and health-related well-being.

To measure the general and personal beliefs in a just world, the studies included in this review used the scales of Dalbert et al. (1999) [15,31,34,36,37,38,39,40,42,44,46,47], Lipkus et al. (1996) [16,32,35], and Lucas et al. (2011) [1]. The constructs that were analyzed to evaluate health-related well-being were self-esteem [31,35,40,42,46], psychological distress [16], life satisfaction [1,15,32,34], anxiety [32,38,40,44], depression [32,37,38,44], stress [32], general well-being [16,36,39,42], and emotional and behavioral symptoms [47] (Figure 2).

The evidence analyzed supports the positive relationship between just-world beliefs and well-being in different populations. General and personal beliefs confer a fairer view of the world and consequently better health outcomes. This positive association was found in all the groups studied [1,15,16,31,32,34,35,36,37,38,39,40,42,44,46,47].

It has been suggested that just-world beliefs can play an important role as a significant psychological resource for children, adolescents, and adults facing adversity [31,32,34,35]. In Greece, a study of a group of primary school students revealed that beliefs in a just world were positively associated with self-esteem [46]. In China, a study that included adolescents recruited from five schools revealed that adolescents who believed that the world was fairer had lower levels of depression [37]. These results are similar to those found in college students. Research with psychology students at an Australian university revealed that just-world beliefs were positively associated with perceived control and self-esteem [35]. Similar findings were obtained at a university in Turkey, where personal beliefs in a just world were positively related to life satisfaction [15]. Additionally, during the coronavirus disease 2019 (COVID-19) pandemic, college students with strong beliefs in justice presented lower levels of academic anxiety [40].

Studies in vulnerable groups have shown similar results in terms of beliefs in a just world and health-related well-being. For example, in Australia, the beliefs in a fair world in women deprived of their liberty and the influence of these beliefs on mental health were evaluated; women who believed that the world treated them more fairly had better well-being and optimistic and resilient attitudes, which translated to low levels of psychological distress [16].

Some studies that have evaluated beliefs in a just world and their relationship with well-being in populations that present certain health problems or disabilities have also found a positive relationship between these aspects. For example, in patients with chronic obstructive pulmonary disease, patients who strongly supported these beliefs were less likely to experience anxiety and depression and were better able to cope with the symptoms of their disease [38]. In cancer patients, just-world beliefs were found to serve as a way of coping with stress related to traumatic events; moreover, just-world beliefs were related to greater acceptance of the disease and better well-being and mental health [39]. Similar findings have been reported in people living with HIV [36] and in people with visual and hearing disabilities [44].

Study findings suggest that both personal and general beliefs in a just world can have positive effects on well-being. However, evidence has shown that personal beliefs play a more relevant role in the perception of well-being than general beliefs do [1,34,42,46]. For example, a study of Thai university students revealed that while personal beliefs were negatively associated with the presence of depressive symptoms, general beliefs were not significantly related to the presence of depressive symptoms [34]. Other studies have shown a positive relationship between both dimensions of just-world beliefs and well-being but to different extents. For example, a study carried out in the United States found a positive and significant relationship between beliefs in a just world (general and personal) and life satisfaction in a general population sample; however, this positive relationship was more pronounced in the case of personal beliefs in a just world [1].

To broaden the understanding of how just-world beliefs relate to well-being, it is essential to explore the mechanisms involved in this connection. Perceived control, hopelessness, self-esteem, resilience, and a sense of power are some of the factors that can explain this relationship [15,16,42]. For example, in a group of Turkish university students, perceived control and hopelessness mediated the positive relationship between just-world beliefs and life satisfaction. In this study, a sense of social justice was positively associated with a sense of perceived control, which resulted in a reduction in feelings of hopelessness and an increase in life satisfaction [15]. In Russian university students, both self-esteem and resilience played a mediating role in the relationship between beliefs in a just world and various indicators of well-being. Self-esteem acted as a mediator in the relationships between just-world beliefs and depressive symptoms, positive and negative affects, and mental well-being. On the other hand, resilience mediated the relationships between just-world beliefs and positive affect and mental well-being but not depressive symptoms or negative affect [42]. In addition, a feeling of power was a significant mediator in the relationship between system justification and subjective well-being, both in persons deprived of liberty and in those who are not [16].

Finally, it has been revealed that beliefs in a just world and health-related well-being can be influenced by a variety of factors, such as socioeconomic status, age, and religiosity. These factors have been noted as predictors of beliefs in a just world and also have a significant influence on health-related well-being [1,37,47]. It is worth exploring these variables as potential moderators of this relationship.

### 3.2. System Justification and Health-Related Well-Being

**Figure 3:**
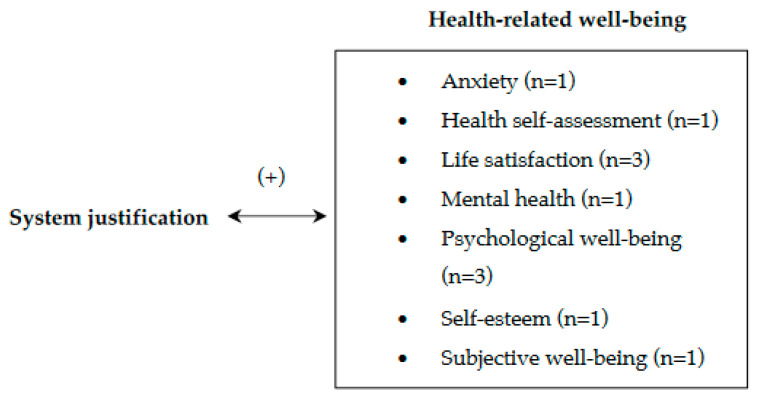
System justification and health-related well-being.

To measure system justification, the studies included in this review used the Kay and Jost scale in different languages [18,19,20,21,22,30,33,41,43,45]. The constructs that were analyzed to evaluate health-related well-being were psychological well-being [18,22,30], life satisfaction [19,30,41], health self-assessment [33], self-esteem [20], anxiety [43], mental health [45], and subjective well-being [21] (Figure 3).

In general, research has revealed that people who show a greater propensity for system justification experience higher levels of health-related well-being. As with just-world beliefs, this positive association has been consistently observed in studies of children, adolescents, and adults in different social contexts [18,19,20,21,22,30,33,41,43,45].

It is important to recognize some of the mechanisms that could influence the positive relationship between system justification and well-being. Among these, the ability of system justification to reduce the perception and impact of discrimination and stigma in certain groups stands out. That is, those individuals who justify the system could develop more effective strategies that would allow them to face discriminatory experiences, which would contribute to an improvement in their well-being [18,21]. For example, a study carried out in Chile with a population of gay and lesbian individuals revealed that minimizing the perception of stigma, that is, minimizing the stigmatizing nature of certain situations, significantly mediated the relationship between system justification and well-being in this population [18]. Similarly, a study carried out in the United States on men who have sex with men showed that the minimization of discrimination against sexual minorities in general could be part of the mechanism that explains the positive relationship between system justification and well-being [21].

Another mechanism that has been associated with the positive effect of system justification is the ability of system justification to generate a sense of belonging. A study of a general population sample showed that a sense of belonging mediated the positive relationship between system justification and general well-being [22]. This finding indicates that as the participants justified the system more, they in turn felt more valued, included, and connected with others, which would confer a feeling of well-being.

Some aspects that could influence the relationship between system justification and health-related well-being are socioeconomic status, gender, educational level, and political orientation. One study evaluated the relationship between system justification and life satisfaction in people of high and low socioeconomic status, showing that system justification significantly predicts life satisfaction in both groups; however, this relationship is more marked among people with lower socioeconomic status. These results could be supported by the capacity of system justification to generate hope for improving the economic situation in the future [19]. Gender could be another variable that moderates the association between system justification and well-being. For example, a study conducted in a youth retention center revealed that men were more likely to justify the system than women. However, it is interesting to note that women who show a strong inclination toward system justification tend to experience fewer mental health problems than men do in those same settings [45]. Other studies have suggested that women who deny gender discrimination and justify the system tend to report greater subjective well-being than men or women who show a lower willingness to justify the system [41,43]. Also, the association between system justification and self-perceived health may be stronger among individuals with higher educational levels compared to those with lower educational levels [33]. Additionally, research indicates that the link between system justification and health-related well-being may be more noticeable among individuals with conservative ideologies than those with less conservative ideologies [18,22,30].

## 4. Discussion

The results of this narrative review demonstrate the positive association of beliefs in a just world and system justification with the perception of well-being [1,15,16,18,19,20,21,22,30,31,32,33,34,35,36,37,38,39,40,41,42,43,44,45,46,47]. These associations were found in a wide variety of groups, including adolescents, ethnic minorities, sexual minorities, and individuals affected by diseases or disabilities, and in the general population in various countries and contexts [1,15,16,18,19,20,21,22,30,31,32,33,34,35,36,37,38,39,40,41,42,43,44,45,46,47]. Together, these findings support the concept of the palliative effect that these ideologies could have. Believing in fairness could help people cope with uncertainty and adversity, providing a sense of control and stability in difficult situations [6,7,12,13].

Particularly in the case of just-world beliefs, the positive relationship of these beliefs with well-being seems to vary depending on whether general or personal beliefs are addressed. In the reviewed studies, it was observed that personal beliefs could have a more marked influence on the well-being of people [1,34,42,46]. A possible explanation for these findings is that personal beliefs are shaped through the unique experiences of each individual, as well as their personal perceptions of justice in specific situations [12,13]. As a result, people interpret their own experiences and events through the lens of what they consider fair and just, which influences their personal beliefs about the fairness of the world. This direct connection between individual experiences and personal beliefs can lead to a stronger and more consistent relationship with well-being than more general beliefs about justice [12,13].

The articles reviewed reveal the complexity of the relationships among the variables studied. Among the analyzed evidence, certain mechanisms were observed through which beliefs in a just world and system justification could influence well-being. In the case of just-world beliefs, the mechanisms analyzed were perceived control, stigma, hopelessness, self-esteem, resilience, and a feeling of power [15,16,36,42]. These study findings suggest that, to the extent that people believe that they live in a just world, their perception that they are capable of influencing their environment increases by maintaining a more positive attitude about the future; their perception of stigma would decrease; their self-esteem would then be strengthened, and they would show greater capacity to adapt and recover from adversity, all of which would increase their perception of wellness [12,13]. Regarding resilience, in our review, two articles showed a positive association between just-world beliefs, resilience, and well-being [16,32]. This association may be particularly relevant in situations of personal dissonance, such as catastrophic illnesses or experiences of discrimination. Reinforcing or restoring these beliefs could foster resilience, alleviate stress, and promote well-being [51].

On the other hand, in the case of system justification, the studies focused on how this type of belief could affect feelings of belonging, discrimination, and stigma [18,21,22]. Those who justify the system are more likely to ignore or minimize situations of stigma and discrimination and feel more strongly valued in a group, which can have positive effects on their well-being [6,7]. The reviewed evidence has shown that there are different mechanisms studied in the context of both ideologies, which likely reflect the particularities of each study. We do not rule out the existence of common mechanisms; for example, stigma might operate similarly in both ideologies [18,36].

Although our review only revealed positive effects, it is important to acknowledge that these ideologies can also have negative impacts on individuals’ well-being. While the personal and general dimensions of just-world beliefs are positively associated, they may serve different psychological functions [11,13,49]. The personal dimension tends to enhance well-being, whereas the general dimension could diminish it or have no effect at all [1,34,52]. Regarding system justification, it may function as a coping mechanism, generating positive effects, but it can also be a source of distress, leading to negative outcomes [53]. Although the previous literature has highlighted contradictory roles, these were scarcely reflected in the findings of this review.

A factor to consider when analyzing the effects of these ideologies on well-being, which may explain the results of this review, is how their impact evolves over time. For instance, the previous literature on system justification suggests that it may have short-term positive effects on well-being, while its long-term impact could be negative [54]. Other researchers have indicated that this positive association may persist over time [4]. Therefore, it is plausible that these ideologies have differential effects on individuals’ well-being over time, yet evidence remains limited [55]. Most studies included in this review are cross-sectional, which only allows for the inference of an initial positive association between these ideologies and well-being. Regarding the longitudinal studies reviewed, the majority did not show significant associations between these variables [22,30,32]. Only one study on adolescents found that system justification was positively associated with self-esteem in the short term but negatively in the long term [20]. These findings could be related to developmental stages from childhood to adulthood, although this remains uncertain [55].

In addition, there are several factors that could explain the degree to which people believe in a just world or justify the system, and some of these factors also have a relationship with health and well-being. From this perspective, these variables would have a moderating role between these ideologies and well-being. These factors include socioeconomic status, educational level, age, religion, gender, and political orientation. People with higher educational and socioeconomic levels tend to justify the system more than those who are more disadvantaged, possibly because people with higher educational and socioeconomic levels have access to more resources and opportunities, which allows them to develop a more equitable perspective of the world and benefit more than disadvantaged groups [1,33,37,47]. With respect to age and religion, it was observed that older people tend to have more ingrained beliefs in justice, possibly due to the accumulated experiences that shape their attitudes on justice and the system. In addition, many religions teach values of justice, equity, and social order, which can influence individual beliefs about justice [1]. With respect to gender, men show a greater tendency to justify the system than women do. Interestingly, despite this greater propensity to justify the system, men report lower levels of well-being in general [45]. This finding suggests a complexity in the relationship between system justification and well-being, and this relationship could be influenced by gender norms, socialization, and differences in access to opportunities in the social system in which people operate [20,30,41,43,45]. Finally, the most conservative people tend to attach great importance to social hierarchy and tradition, highlighting the importance of adhering to established social norms and showing respect for authority [5,18,22,30].

## 5. Future Considerations

Several of the findings of this review allow us to identify possible areas of focus in future research on the associations of beliefs in a just world and system justification with people’s health-related well-being.

First, given the narrative nature of this article, future research could explore differences in effect sizes between these constructs through a meta-analysis. Although we provide an overview of these relationships, a meta-analysis could offer a more detailed quantitative assessment, highlighting consistent patterns and facilitating a clearer understanding of the magnitude and direction of the effects, which could enrich and strengthen this topic.

In this literature review, different mechanisms through which the ideologies addressed would favor well-being were identified. Owing to the theoretical proximity of these ideologies, it would be interesting to demonstrate the degree to which these mechanisms could be common to both ideologies. Identifying the points of convergence between these ideologies would allow us to understand more deeply how these ideologies interact to promote health-related well-being.

The scant literature on the relationship of religion with just-world beliefs suggests that this is an area that has been insufficiently explored in research. The fact that only one article has addressed this topic highlights the need for broader and more exhaustive research in this area [1]. The positive relationship observed between religiosity and just-world beliefs suggests that religious people may have a unique perspective on inequality, possibly influenced by their religious values, principles, and teachings. Exploring these differences further could lead to a more in-depth understanding of how religion influences subjective well-being and the perception of society in general.

Finally, there are parts of the world where research in this line has been more widely conducted, and future research should address these ideologies in less developed countries or in those with important social and economic changes that could influence people’s well-being. To date, there has been a lack of attention to the understanding and meaning of these associations, as well as their possible health implications, for people in some parts of the world, such as Latin America. Contextual factors can play a crucial role in the functioning of psychological mechanisms related to social justice [9]. Recent studies have revealed that people who live in more developed countries and justify the system experience less anxiety and depression. Similarly, countries with higher gross domestic product (GDP), higher educational levels, and longer life expectancies are also associated with greater well-being among residents [4]. An underperforming social system or a less developed country could threaten beliefs in a just world, which, consequently, would translate into a poorer sense of well-being [9].

## 6. Conclusions

This narrative review reveals how beliefs in a just world and system justification are positively associated with people’s health-related well-being. This relationship is observed in different demographic and cultural groups, which supports the hypothesis that both ideologies could have palliative effects.

## Figures and Tables

**Table 1:** Search strategy.

Database	Key Words	Search Strategy	Year
WoS	Health, well-being, anxiety, mental health, physical health, quality of life, stress, self-esteem, life satisfaction, just world, and system justification	HEALTH (Topic) or WELLBEING (Topic) or ANXIETY (Topic) or DEPRESSION (Topic) or “MENTAL HEALTH” (Topic) or “PHYSICAL HEALTH” (Topic) or “QUALITY OF LIFE” (Topic) or STRESS (Topic) or “SELF-ESTEEM” (Topic) or “LIFE SATISFACTION” (Topic) AND “JUST WORLD” (Topic) Or “SYSTEM JUSTIFICATION”	2019–2023
Scopus	Health, well-being, anxiety, mental health, physical health, quality of life, stress, self-esteem, life satisfaction, just world, and system justification	((TITLE-ABS-KEY (health) OR TITLE-ABS-KEY (wellbeing) OR TITLE-ABS-KEY (anxiety) OR TITLE-ABS-KEY (“MENTAL HEALTH”) OR TITLE-ABS-KEY (“PHYSICAL HEALTH”) OR TITLE-ABS-KEY (“QUALITY OF LIFE”) OR TITLE-ABS-KEY (stress) OR TITLE-ABS-KEY (self-esteem) OR TITLE-ABS-KEY (“LIFE SATISFACTION”))) AND ((TITLE-ABS-KEY (“just world”) OR TITLE-ABS-KEY (“system justification”)))	2019–2023

Note: own elaboration.

**Table 2:** Eligible articles for the narrative review.

Authors	Objective	Year	Population	Country	Ideology	Well-Being Constructs	Principal Results
Bahamondes et al. [18]	To examine whether the beliefs justifying the system confer palliative benefits to the well-being of sexual minorities by minimizing stigma and sexual discrimination.	2020	Gays and lesbians (*n* = 467)	Chile	System justification ^a^	Psychological well-being	System justification was negatively associated with psychological distress via minimizing perceptions of sexual stigma towards the ingroup.
Bahamondes et al. [22]	To examine the associations that approach and avoidance relational goals have with system justification.	2021	General sample (*n* = 21,936)	New Zealand	System justification ^a^	Psychological well-being	Avoidance goals correlated positively with system justification. Sequential mediation analyses revealed that avoidance goals predicted higher well-being via system justification and belongingness.
Bahamondes et al. [30]	To evaluate the mediation role of group-based discrimination in the relationship between system-justifying beliefs and well-being.	2019	General sample (*n* = 12,959)	New Zealand	System justification ^a^	Psychological well-being and life satisfaction	The ethnic minorities and women generally report lower levels of well-being than New Zealand Europeans and men do, respectively. These differences were mitigated by the endorsement of ethnic- and gender-specific system justification, respectively. Mediated moderation analyses further revealed that part of the palliative effects of system justification occurred via reductions in perceived group-based discrimination.
Bai et al. [31]	To investigate the relationship between cyberbullying victimization and suicidal ideation among adolescents.	2021	Adolescents (*n* = 3322)	China	Just-world beliefs ^b^	Self-esteem	The results indicated a positive correlation between cyberbullying victimization and suicide ideation, which was mediated by belief in a just world. Self-esteem and perceived social support moderated the negative correlation between cyberbullying victimization and belief in a just world.
Bartholomaeus and Strelan [16]	To investigate differences between prisoners and non-prisoners in their endorsement of personal beliefs in a just world, levels of empowerment, well-being, optimism, resilience, and psychological distress.	2021	Prisoners (*n* = 72) and non-prisoners (*n* = 80)	Australia	Just-world beliefs ^c^	General well-being and psychological distress	Personal BJW functions through a sense of power to promote adaptive psychological function similarly for prisoner and non-prisoner populations. Prisoners reported higher levels of psychological distress than non-prisoners. Prisoners reported higher levels of resilience compared to non-prisoners. Prisoners reported non-significantly higher levels of well-being and lower levels of optimism. Personal BJW functions in a similar empowering way to promote positive outcomes.
Bartholomaeus et al. [32]	To test whether the empowering function of personal beliefs in a just world generalizes to a variety of well-being outcomes.	2023	Undergraduate students (*n* = 307) and general sample (*n* = 450)	Australia	Just-world beliefs ^c^	Anxiety, depression, stress, and life satisfaction	There was a positive indirect effect of personal BJW on life satisfaction, optimism, and resilience through empowerment. Similarly, personal BJW has a negative indirect effect on depression, anxiety, and stress through empowerment.
Card and Hepburn [33]	To explore which dimensions of social position facilitate the rejection of system justification beliefs and which are associated with higher levels of system justification.	2022	General sample (*n* = 2619)	Canada	System justification ^a^	Health self-assessment	Social marginalization is associated with less system justification. Those benefitting from the status quo were more likely to hold system-justifying beliefs. The multivariable results showed that higher economic system justification scores were associated with lower levels of loneliness and higher self-rated physical health.
Chobthamkit et al. [34]	To examine the relationships between personal and general beliefs in a just world, life satisfaction, and depression in Thailand and the United Kingdom.	2022	Undergraduate students (*n* = 697)	Thailand and the United Kingdom	Just-world beliefs ^b^	Life satisfaction and depression	In both studies, personal BJW uniquely predicted well-being. When controlling for BJW, belief in karma positively predicted life satisfaction and depression only in the UK sample. In addition, karma was uniquely predicted by general BJW but more strongly so in Thailand. Furthermore, within both samples, individuals endorsed personal BJW more strongly than general BJW.
Collins and Strelan [35]	To test the interaction between beliefs in a just world and self-esteem.	2021	Psychology students (*n* = 131) and general sample (*n* = 67)	Australia	Just-world beliefs ^c^	Self-esteem	Individuals who value fairness but score low on just-world beliefs for the self tend to indicate reduced self-esteem. In contrast, this effect does not occur when individuals who value fairness score high on BJW-self. That fairness has an indirect effect on self-esteem through intrapersonal consistency, and BJW-self has an indirect effect on self-esteem through personal control.
Đorić SN. [36]	To explore the relationship between domains of HIV-related stigma, just-world beliefs and well-being in people living with HIV.	2020	People living with HIV (*n* = 90)	Serbia	Just-world beliefs ^b^	General well-being	A significant relationship emerged between the domains of stigma and the components of subjective well-being, which is not direct but is rather mediated by belief in a just world. The findings indicate that exposure to stigma can lead to a decrease in belief in a just world, which potentially leads to a sense of lack of control over one’s life, with a final, negative outcome for subjective well-being.
Feng, N., Xie, Z., Li, Y. et al. [37]	To examine the mediation role of just-world beliefs in the relationship between stressful childhood environments and depression among adolescents.	2023	Adolescents (*n* = 3553)	China	Just-world beliefs ^b^	Depression	Results showed that childhood unpredictability and harshness were both positively associated with adolescent depression. The serial mediation analysis suggested that adolescents with a higher experience of stressful childhood environments perceived more discrimination, which was related to lower belief in a just world and subsequently associated with higher adolescent depression.
Godfrey et al. [20]	To examine how beliefs about the fairness of the U.S. system in sixth grade influence self-esteem and behavioral trajectories among teenagers.	2019	Adolescents (*n* = 257)	United States	System justification ^a^	Self-esteem	System justification was associated with higher self-esteem, less delinquent behavior, and better classroom behavior in sixth grade but worse trajectories of these outcomes from sixth to eighth grade.
Harding et al. [1]	To explore the relationship between the four subscales of beliefs about a just world and life satisfaction overall.	2020	General sample (*n* = 301)	United States	Just-world beliefs *^d^*	Life satisfaction	Respondents who identified themselves as middle and upper class reported higher levels of life satisfaction than those who identified themselves as lower class, with a medium effect size. Regressing life satisfaction on the four justice beliefs subscales (distributive justice for others, procedural justice for others, distributive justice for self, procedural justice for self) indicated that the two self-subscales were significantly predictive of life satisfaction, but the two other subscales were not.
Jian et al. [38]	To explore the effects of personal and general beliefs about a just world on patients’ mental health.	2021	General sample (*n* = 147)	China	Just-world beliefs ^b^	Anxiety and depression	For patients with low personal BJW, lower health-related quality of life was correlated with higher depression. For patients with stronger endorsement of general BJW, worse health-related quality of life was associated with higher depression and anxiety, but the variance in anxiety caused by the interaction was insignificant.
Kiral Ucar et al. [15]	To investigate the mediating role of perceived control and hopelessness in the relationship between personal belief in a just world and life satisfaction.	2019	Undergraduate students (*n* = 354)	Turkey	Just-world beliefs ^b^	Life satisfaction	Personal BJW was significantly associated with increased life satisfaction after controlling for perceived control and hopelessness. Further, both perceived control and hopelessness uniquely mediated the association between personal BJW and life satisfaction. Personal BJW tended to increase life satisfaction uniquely through both increased perceived control and decreased hopelessness. Finally, personal BJW increased perceived control, which in turn decreased hopelessness and subsequently increased life satisfaction.
Li et al. [19]	To examine whether system justification enhances psychological well-being among both favored and disfavored group members.	2020	Study 1: general sample (*n* = 10,196); study 2: adolescents (*n* = 4037); and study 3: undergraduate students (*n* = 172)	China	System justification ^a^	Life satisfaction	System justification positively predicts both high-class and low-class individuals’ life satisfaction, and this result holds for both adults and adolescents. System justification has a causal effect on life satisfaction through an increased level of perceived individual upward mobility.
Megías et al. [39]	To understand the relationships between beliefs about a just world and emotional intelligence and the well-being and quality of life of cancer patients.	2019	Cancer patients (*n* = 68)	Spain	Just-world beliefs ^b^	General well-being	Different multiple regression analyses showed that patients’ personal BJW negatively predicted their anxiety and a trend to a better quality of life. In addition, patients with high scores in the mood repair subfactor of emotional intelligence showed better quality of life, and those with higher attention to feelings exhibited more anxiety and a trend to more depression.
Na and Tian [40]	To explore the connection between personal beliefs about a just world and academic anxiety among university students in the context of the COVID-19 pandemic.	2023	Undergraduate students (*n* = 96)	China	Just-world beliefs ^b^	Anxiety and self-esteem	A participant’s low level of a personal BJW may serve as an early predictor to identify students with academic anxiety. In addition, high correlations among physical activity, a personal BJW, and academic anxiety were identified, and the specified moderate physical activity, doing household chores, may serve as an early intervention to help college students manage academic anxiety.
Napier et al. [41]	To understand why individuals, especially women, might deny the existence of gender discrimination from a system justification perspective.	2020	General sample (*n* = 793)	Multinational	System justification ^a^	Life satisfaction	That denial (vs. acknowledgement) of gender discrimination is associated with higher subjective well-being among women, and this is because denying gender discrimination promotes the view that the system is fair. We further show that this happens above and beyond personal experiences with sexism and that the association is stronger in countries where sexism is relatively high (vs. low).
Nartova-Bochaver et al. [42]	To investigate how personal and general beliefs of a just world could predict subjective well-being in students.	2019	Undergraduate students (*n* = 627)	Russia	Just-world beliefs ^b^	Self-esteem and general well-being	The findings show that personal BJW is related to all investigated indicators of well-being. Self-esteem mediated all relations between personal BJW and indicators of subjective well-being, whereas resilience mediated relations of personal BJW to positive affect and mental well-being. The pattern of results persisted when we controlled for age, gender, religiosity, and general BJW.
Pacilli et al. [43]	To examine whether system justification motivation interacts with exposure to workplace sexism and how it affects the psychological adjustment of women.	2019	Portuguese women (*n* = 92)	Portugal	System justification ^a^	Anxiety	The results indicated that both hostile sexism and benevolent sexism fostered participants’ anxiety and that system justification moderated the relation between hostile sexism and anxiety. Anxiety was highest among participants low in system justification.
Pojatic and Degmecic [44]	To determine depression levels and anxiety symptoms in blind and deaf individuals based on beliefs about a just world.	2019	Deaf and blind people (*n* = 133)	Croatia	Just-world beliefs ^b^	Anxiety and depression	Blind participants had stronger beliefs in a just world in comparison with deaf participants and participants in the control group. On the other hand, there were no significant differences between deaf participants and the control group. Among the deaf participants, the stronger their belief in a just world, the lower their level of anxiety was. Among the participants in the other two groups, there were no statistically significant correlations between the expressed beliefs in a just world and symptoms of anxiety and depression.
Sichel et al. [45]	To examine associations between specific mental health outcomes and system justification in a sample of young people involved in the legal system.	2022	Adolescents (*n* = 196)	United States	System justification ^a^	Mental health	Results suggested that boys were more likely to endorse societal fairness compared to girls, but these beliefs were unrelated to their mental health. However, a significant gender moderation was found such that girls who perceived society to be fair reported lower levels of internalizing and externalizing mental health problems.
Suppes et al. [21]	To examine the perception of discrimination among LGBT people and its relationship with well-being.	2019	Gays and bisexuals (*n* = 133)	United States	System justification ^a^	Subjective well-being	Results show those who minimize (vs. acknowledge) the extent to which their group is the target of discrimination report better well-being across myriad indicators. It was also demonstrated that this effect is mediated by perceived system fairness; holds above and beyond internalized homonegativity and ingroup identification; and is true regardless of whether individuals reside in hostile or accepting environments and regardless of whether individuals had personally experienced discrimination.
Tatsi and Panagiotopoulou [46]	To examine self-esteem levels, personal beliefs, and general beliefs about a just world in elementary school students.	2023	Elementary school students (*n* = 292)	Greece	Just-world beliefs ^b^	Self-esteem	The results indicated that students more strongly endorse personal BJW than general BJW. Also, personal BJW and children’s place of residence significantly predict self-esteem, indicating that the more the students feel they are treated fairly, the better their level of self-esteem.
Weinberg et al. [47]	To investigate the existence of a social gradient in the mental health of adolescents in the Netherlands.	2023	Adolescents (*n* = 848)	The Netherlands	Just-world beliefs ^b^	Emotional and behavioral symptoms	Adolescents with lower family affluence and lower perceived family wealth reported more emotional symptoms, and the association between perceived family wealth and emotional symptoms was mediated by lower personal and general BJW. Furthermore, higher personal BJW amplified the negative association between socioeconomic status and peer problems.

Note: own elaboration; ^a^ general system justification scale (Kay and Jost) [48]; ^b^ beliefs in a just world scale (Dalbert et al.) [12]; ^c^ beliefs in a just world scale (Lipkus et al.) [49]; ^d^ the belief in a just world scale (Lucas et al.) [50].
